# The association of the dietary inflammatory potential with risk of overall and site-specific cancers: A community-based longitudinal study in the UK Biobank

**DOI:** 10.1016/j.jnha.2024.100225

**Published:** 2024-04-05

**Authors:** Jiaxin Liang, Rongrong Yang, Huiying Da, Jiao Wang, Maiwulamujiang Maimaitiyiming, Xiuying Qi, Michelle M. Dunk, Weili Xu

**Affiliations:** a Department of Epidemiology and Biostatistics, School of Public Health, Tianjin Medical University, Tianjin, China; b Tianjin Key Laboratory of Environment, Nutrition and Public Health, Tianjin, China; c Center for International Collaborative Research on Environment, Nutrition and Public Health, Tianjin, China; d Public Health Science and Engineering College, Tianjin University of Traditional Chinese Medicine, Tianjin, China; e Department of Epidemiology, College of Preventive Medicine, Third Military Medical University, Chongqing, China; f Aging Research Center, Department of Neurobiology, Health Care Sciences and Society Karolinska Institutet and Stockholm University, Stockholm, Sweden

**Keywords:** The dietary inflammatory potential, Cancer risk, Overall cancer, Site-specific cancer, UK Biobank

## Abstract

**Objectives:**

The association of the dietary inflammatory potential with cancer risk remains uncertain. We examined the relationship of the dietary inflammatory potential with risk of overall and site-specific cancers and explored its sex and age differences.

**Design:**

A community-based longitudinal study.

**Setting:**

Participants from the UK Biobank completed baseline surveys during 2006–2010 and were followed for up to 15 years to detect incident cancer.

**Participants:**

170,899 cancer-free participants with dietary data available (mean age: 55.73 ± 7.95, 54.10% female).

**Measurements:**

At baseline, dietary intake was assessed with a 24-h dietary record for up to 5 times. The inflammatory diet index (IDI) was calculated to assess the dietary inflammatory potential as a weighted sum of 31 food groups (including 14 anti-inflammatory and 17 pro-inflammatory) based on plasma high-sensitivity C-reactive protein (hsCRP) levels, and tertiled as low (indicating low-inflammatory diet), moderate, and high IDI (as reference). Overall and site-specific cancers were ascertained via linkage to routine hospital admission, cancer registry, and death certificate data. Data were analyzed using Cox regression and Laplace regression.

**Results:**

During the follow-up (median 10.32 years, interquartile range: 9.95–11.14 years), 18,884 (11.05%) participants developed cancer. In multi-adjusted Cox regression, low IDI scores were associated with decreased risk of rectal cancer (hazard ratio [95% confidence interval, CI] 0.76 [0.61, 0.94]), thyroid cancer [0.45 (0.27, 0.74)], lung cancer [0.73 (0.61, 0.88)]. However, the association between IDI score and the risk of overall cancer was not significant. Laplace regression analysis showed that 10th percentile differences (95% CIs) of cancer onset time for participants with low IDI scores was prolonged by 1.29 (0.32, 2.27), 1.44 (0.58, 2.30), and 2.62 (0.98, 4.27) years for rectal cancer, thyroid cancer, and lung cancer, respectively, compared to those with high IDI scores. Stratified analysis revealed that low IDI scores were associated with a lower risk of rectal cancer (p interaction between IDI score and sex = 0.035) and lung cancer in males, but not in females, and with a reduced risk of thyroid cancer in females, but not in males. Moreover, low IDI scores were associated with a reduced risk of rectal cancer and lung cancer in the participants aged ≥60 years, but not in those <60 years, and with a reduced risk of thyroid cancer in those aged ≥60 years and <60 years.

**Conclusions:**

A low-inflammatory diet is associated with decreased risk and prolonged onset time of rectal cancer and lung cancer, especially among males and individuals aged ≥60 years, and thyroid cancer among females.

## 1 Introduction

According to the latest global cancer burden data released by the International Agency for Research on Cancer (IARC), there were an estimated 19.3 million incident cancer cases and 10 million cancer deaths worldwide in 2020 [1]. Additionally, the combined cancer incidence rate for men (222.0 per 100,000) was 19% higher than for women (186 per 100,000) [1]. Both cancer and cancer treatment can cause physical, mental, and financial distress for patients and their families. Cancer also creates societal burdens by increasing national healthcare expenditures [2]. It has been estimated that up to half of all cancers are preventable through primary prevention by limiting and improving risk factors, many of which are modifiable through lifestyle behaviors [3].

Diet has been identified as one modifiable risk factor that can play a critical role in cancer prevention [4]. Unhealthy diets are associated with a higher risk of cancer [5]. In 2017, more than 11 million people died from unhealthy diets, and more than 930,000 of these deaths were due to cancer, particularly breast and colorectal cancers [4]. However, the specific mechanisms by which diet affects cancer development are not well understood. A growing number of researchers have focused on the association of dietary inflammatory potential with cancer, but results have been inconsistent. Some studies found that high dietary inflammatory potential was associated with increased risk of overall [6], breast [7], rectal [8], liver [9], and lung cancers [10], while other studies did not report such associations [[11], [12], [13], [14], [15], [16]].

The incidence of cancer and the impact of diet on cancer may differ by sex and age due to differences in genetic, epigenetic, hormonal, and environmental factors [17]. However, few of the previous studies on the association between inflammatory properties of the diet and cancer have explored sex or age differences. Among the studies on inflammatory properties of the diet and lung cancer risk, only one case-control study was stratified by gender, which found that a pro-inflammatory diet was associated with increased odds of lung cancer in men, but not women [18].

In order to identify potential cancer risk reduction strategies, there is a need for further investigation of the relationship between dietary inflammatory potential and cancer risk, and whether this association varies by sex or age. In the current study, we aimed to: 1) examine the association between dietary inflammatory potential and the risk of overall cancer and site-specific cancers; and 2) explore sex and age differences in such association among UK Biobank participants.

## 2 Materials and methods

### 2.1 Study population

**Fig. 1:**
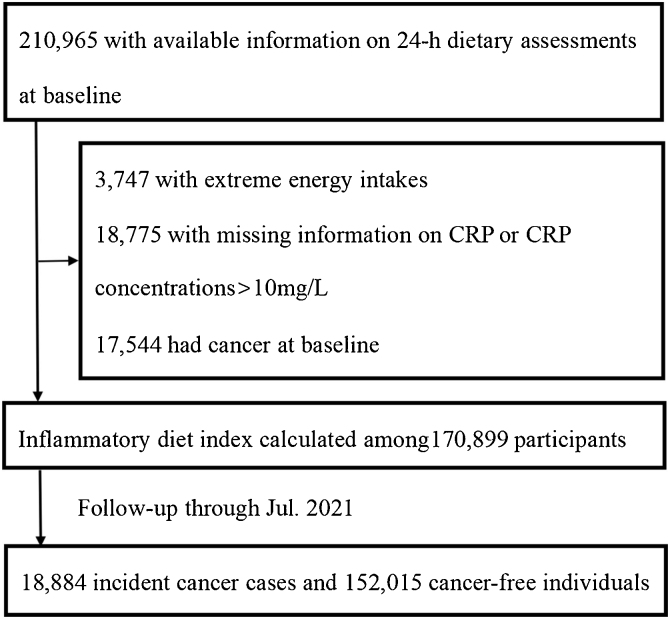
Flowchart of the study population.

The UK Biobank is a community-based longitudinal study. From 2006 to 2010, 502,412 UK residents aged 37–73 were recruited from 22 research centers (England, Wales, and Scotland) in the baseline survey. Among them, 210,965 participants completed at least one 24-h dietary assessment, we progressively excluded 3747 participants with extreme energy intake (men: <800 or >4200 kcal/day; women: <600 or >3500 kcal/day), 18,775 with missing information on plasma high-sensitivity C-reactive protein (hsCRP) or outlier values of hsCRP concentrations (>10 mg/L), and 17,544 with prevalent cancer at baseline, leaving 170,899 participants included in the final analysis (Fig. 1).

### 2.2 Data collection

At baseline, information on age, sex, education level, race, socioeconomic status, smoking status, physical activity, and medical history was collected through interviews and touchscreen questionnaires. Weight and height were measured, and peripheral blood samples were collected for biochemical assays. Education level was categorized as college or non-college based on the highest level of formal education attained. Race was categorized as white or non-white (including Asian, Black, Chinese, and Mixed). Socioeconomic status was determined by the Townsend deprivation index (encompassing information on social class, employment, car availability, and housing) [19] and categorized as low (highest quintile), middle (quintiles 2–4), and high (lowest quintile) [20]. Smoking status was divided into three categories: never, previous, or current. Physical activity was categorized as regular or irregular physical activity. Regular physical activity was defined as engaging in either ≥150 min moderate activity per week, ≥75 min vigorous activity per week or equivalent combination, moderate physical activity at least 5 days a week, or vigorous activity once a week [21]. Body mass index (BMI) was calculated as weight (kg) divided by height squared (m^2^) and was categorized as <20 kg/m^2^ (underweight), 20–24.9 kg/m^2^ (normal weight), 25–29.9 kg/m^2^ (overweight), or ≥30 kg/m^2^ (obese). Diabetes was ascertained on the basis of medical records (International Classification of Diseases, ICD-10 codes E10 to E14), glycated hemoglobin ≥6.5%, the use of antidiabetic drugs, and a self-reported history of diabetes. Hypertension was defined as a self-reported history of hypertension, systolic blood pressure ≥140 mmHg, diastolic blood pressure ≥90 mmHg, use of anti-hypertensive drugs, or medical records (ICD-10 codes I10 to I13 and I15). Information on other diseases were derived from medical records: I50 for heart failure, I25 for coronary artery disease, I48 for atrial fibrillation and I60–I64, I69 for stroke. All disease states were defined in combination with self-reported and ICD codes. Additional information collected at baseline included age at menarche, age at menopause, number of live births, oral contraceptive use, and use of hormone replacement therapy.

### 2.3 Dietary assessment

Dietary data was obtained at recruitment through the Oxford WebQ questionnaire which included consumption information of 206 types of foods and 32 types of drinks in the past 24 h [22]. Moreover, participants were invited via e-mail to complete an online 24-h dietary assessment every 3–4 months from February 2011 to June 2012 for a total of four times [23]. The average daily quantity consumed of each food or drink was calculated by multiplying the standard portion size by the amount consumed. Total energy intake was calculated using the food composition table of the UK Nutrient Databank [24].

### 2.4 Assessment of inflammatory marker

Plasma hsCRP concentration was measured at baseline by immunoturbidimetry using the Beckman Coulter AU5800. Detailed information on sample collection and processing has been described previously [25]. Given that acute infection, trauma, or medication use during blood sample collection may introduce bias, we excluded participants with plasma hsCRP concentrations greater than 10 mg/L [26]. Plasma hsCRP concentrations were ln-transformed due to skewed distribution.

### 2.5 Assessment of cancers

Overall and site-specific cancers were ascertained via linkage to routine hospital admission, cancer registry, and death certificate data. Incident cancer cases and onset dates were ascertained through hospital admission records linked to Health Episode Statistics (England and Wales) and the Scottish Morbidity Record 01 (SMR01) (Scotland), which can be found at http://content.digital.nhs.uk/services. Cancer cases and specific sites were defined through the diagnostic code according to ICD codes) (Table S1). For participants with multiple cancer diagnoses during follow-up, the type of cancer that first occurred was ascertained according to the earliest recorded date of cancer.

### 2.6 Statistical analysis

#### 2.6.1 Identification of a low-inflammatory diet

As previously described [27], the inflammatory potential of participants’ diet was evaluated by calculating the inflammatory diet index (IDI). First, we calculated the average daily intake of 39 food groups from the Oxford WebQ questionnaire [28] (Table S2).

Second, reduced-rank regression (RRR) was performed to obtain a dietary pattern related to hsCRP concentration (Table S3). In subsequent analyses, we maintained the first factor obtained from the RRR of all 39 food groups (We called this the RRR dietary pattern). Third, stepwise linear regression was performed to identify the food groups that played an important role in the dietary pattern in RRR (p < 0.05). A total of 31 food groups (Table S4) were retained, including 14 anti-inflammatory foods (wine, bread, starch, breakfast cereal, vegetables, fruit, juice, tea, fish, cheese, pastry, desserts, nuts, vegetarian protein alternatives) and 17 pro-inflammatory foods (beer, other alcohol, butter, potatoes, low-calorie drinks, high-caloriedrinks, smoothies, milk, processed meat, red meat, poultry, organ meat, other meat, eggs, ice cream, sweets, chocolate drinks). Finally, each participant’s IDI score was calculated by weighting the sum of the intake of each food group according to the regression coefficient obtained by stepwise linear regression. The IDI score was further tertiled into low, moderate, and high scores representing low-, moderate-, and high-inflammatory diets, respectively.

#### 2.6.2 Data analysis

Baseline characteristics of participants were compared according to IDI score, and differences among the IDI groups were tested using one-way analysis of variance (ANOVA) or Kruskal–Wallis tests for continuous variables, and Chi-square tests for categorized variables.

Cox proportional hazards regression models were used to estimate the hazard ratios (HRs) and 95% confidence intervals (CIs) for the risk of overall cancer and site-specific cancer in relation to IDI score. Censoring was defined as the time of death, withdrawal from the study, or the end of follow-up (July 1, 2021), whichever came first. Stratified analyses were performed to explore differences in IDI-cancer associations by sex (males vs. females) and age (<60 vs. ≥60). To assess multiplicative interactions, we included the cross-product term of IDI and sex/age in the model. Based on the cancer incidence of 11.05% during follow-up period in this study, the 10th percentile differences (PDs) and 95% CIs of cancer onset time in relation to IDI were estimated using Laplace regression model. IDI score was examined both as a continuous variable (per 1 standard deviation [SD] decrease in IDI score) and a categorical variable (high IDI as reference). All analyses included crude, basic-adjusted (by age and sex) and multivariable-adjusted models (by age, sex, race, education, Townsend deprivation index, total energy intake, smoking, physical activity, BMI, diabetes, hypertension, coronary artery disease, atrial fibrillation, heart failure, and stroke). Additional adjustments were made for age at menarche, age at menopause, number of live births, use of oral contraceptives, and use of hormone replacement therapy in the multivariable model of female-specific cancers.

Missing values for education (n = 733), race (n = 543), Townsend deprivation index (n = 203), smoking (n = 372), physical activity (n = 7864), and BMI (n = 386) were multiple imputed using chained equations. In supplementary analyses, we repeated the main analyses after excluding participants with missing values for covariates and excluding cancer cases diagnosed in the first 2 years of follow-up, respectively. R version 4.2.1 and SAS 9.4 (SAS institute, Cary, NC) were used for all statistical analysis. Two-sided p-values <0.05 were considered statistically significant.

## 3 Results

### 3.1 Characteristics of the study population at baseline

The mean age of participants (N = 170,899) at baseline was 55.73 ± 7.95 years (ranging from 39 to 72 years), and 54.1% of participants were female. The majority of participants were white (91.2%). Compared to participants with a high IDI score, those with a low IDI score were more likely to be older, female, non-white, and to have a higher education level and lower BMI. Those with a low IDI score were also more likely to engage in regular physical activity, and less likely to smoke and have diabetes, hypertension, coronary artery disease, atrial fibrillation, heart failure, and stroke (Table 1).

**Table 1:** Baseline characteristics of the study population (N = 170,899) by the inflammatory diet index (IDI).

Characteristics	IDI^a^			*P* value
	High (n = 56,966)	Moderate (n = 56,966)	Low (n = 56,967)	
Age (years)	55.21 ± 8.10	56.11 ± 7.91^b^	55.88 ± 7.81^b^	<0.001
Female	26,925 (47.3)	31,970 (56.1)^b^	33,560 (58.9)^b^	<0.001
Education				<0.001
Non-college	38,186 (67.0)	32,222 (56.6)^b^	26,604 (46.7)^b^	
College	18,780 (33.0)	24,744 (43.4)^b^	30,363 (53.3)^b^	
Race				<0.001
White	52,689 (92.5)	52,253 (91.7)^b^	50,940 (89.4)^b^	
Nonwhite	4277 (7.5)	4713 (8.3)^b^	6027 (10.6)^b^	
Townsend deprivation index	−2.29 (−3.69, 0.16)	−2.43 (−3.79, −0.21)^b^	−2.26 (−3.71, 0.13)^b^	<0.001
Total energy intake (kcal/day)	2089.12 ± 593.26	2019.86 ± 535.54^b^	2148.18 ± 551.49^b^	<0.001
Smoking status				<0.001
Never	30,702 (53.9)	33,646 (59.1)^b^	33,265 (58.4)^b^	
Previous	20,199 (35.5)	19,370 (34.0)^b^	20,431 (35.9)	
Current	6065 (10.6)	3950 (6.9)^b^	3271 (5.7)^b^	
Regular physical activity^c^	39,355 (69.1)	41,840 (73.4)^b^	44,464 (78.1)^b^	<0.001
BMI (kg/m^2^)	28.00 ± 4.77	26.62 ± 4.26^b^	25.79 ± 4.05^b^	<0.001
<20	829 (1.5)	1497 (2.6)^b^	2221 (3.9)^b^	<0.001
20–24.9	15,105 (26.5)	20,503 (36.0)^b^	24,801 (43.5)^b^	
25–29.9	24,863 (43.6)	24,495 (43.0)	22,220 (39.0)^b^	
≥30	16,169 (28.4)	10,471 (18.4)^b^	7725 (13.6)^b^	
Diabetes	3242 (5.7)	2046 (3.6)^b^	1629 (2.9)^b^	<0.001
Hypertension	31,022 (54.5)	28,211 (49.5)^b^	26,151 (45.9)^b^	<0.001
Coronary artery disease	2871 (5.0)	2152 (3.8)^b^	1871 (3.3)^b^	<0.001
Atrial fibrillation	910 (1.6)	785 (1.4)^b^	725 (1.3)^b^	<0.001
Heart failure	283 (0.5)	165 (0.3)^b^	139 (0.2)^b^	<0.001
Stroke	745 (1.3)	544 (1.0)^b^	491 (0.9)^b^	<0.001

Data are presented as mean ± SD, median (IQR), or n (%).

a High: 0.352 ≤ IDI < 10.074, moderate: −0.409 ≤ IDI < 0.352, low: −5.002 < IDI ≤ −0.409.

b Compared to the high IDI group, the difference in basic characteristics was statistically significant.

c Defined as engaging in either ≥150 min moderate activity per week, ≥75 min vigorous activity per week or equivalent combination, moderate physical activity at least 5 days a week, or vigorous activity once a week.

### 3.2 Association between a low-inflammatory diet and cancer

A total of 18,884 (11%) participants developed cancer during follow-up (median 10.32 years, interquartile range: 9.95–11.14 years). The results of multi-adjusted Cox regression models show that low IDI scores (vs. high IDI) were associated with 14%, 26%, and 55% decreased risk of digestive cancers (HR = 0.86, 95%CI: 0.78, 0.95), respiratory cancers (HR = 0.74, 95%CI: 0.61, 0.89), and thyroid cancer (HR = 0.45, 95%CI: 0.27, 0.74), respectively. However, there was no association between IDI and overall cancer. Only low risk of rectal cancer among digestive cancers was related to low IDI scores (low vs. high, HR = 0.76, 95%CI: 0.61, 0.94), while only low risk of lung cancer was related to low IDI scores (HR = 0.73, 95%CI: 0.61, 0.88) among respiratory cancers (Table 2). Results for all other cancers with very few cases are shown in Table S5.

**Table 2:** Hazard ratios (HRs) and 95% confidence intervals (CIs) of overall and site-specific cancer in relation to the inflammatory diet index (IDI).

Cancer	Number of cases	IDI-categorical			P for trend
		High	Moderate	Low	
Overall	18,884	1.00 (Ref)	0.99 (0.96, 1.03)^a^	0.95 (0.92, 0.98)^a^	0.003
		1.00 (Ref)	0.96 (0.93, 0.99)b1, *	0.94 (0.91, 0.97)b1, *	<0.001
		1.00 (Ref)	0.98 (0.94, 1.02)c1	0.97 (0.93, 1.00)c1	0.072
Lip and oral	179	1.00 (Ref)	0.71 (0.49, 1.03)^a^	0.99 (0.70, 1.40)^a^	0.943
		1.00 (Ref)	0.72 (0.49, 1.05)b1	1.02 (0.72, 1.44)b1	0.923
		1.00 (Ref)	0.75 (0.52, 1.10)c1	1.10 (0.77, 1.58)c1	0.614
**Digestive**	2569	1.00 (Ref)	**0.89 (0.82, 0.98)**^a^, *	**0.77 (0.70, 0.85)**^a^, *	**<0.001**
		1.00 (Ref)	**0.88 (0.80, 0.96)**b1, *	**0.78 (0.71, 0.86)**b1, *	**<0.001**
		1.00 (Ref)	**0.94 (0.86, 1.03)**c1	**0.86 (0.78, 0.95)**c1, *	**0.003**
Esophagus	268	1.00 (Ref)	0.76 (0.57, 1.00)^a^	0.55 (0.40, 0.74)^a^	<0.001
		1.00 (Ref)	0.77 (0.58, 1.02)b1	0.59 (0.43, 0.80)b1	<0.001
		1.00 (Ref)	0.91 (0.68, 1.20)c1	0.77 (0.56, 1.05)c1	0.104
Stomach	139	1.00 (Ref)	1.08 (0.73, 1.61)^a^	0.86 (0.57, 1.31)^a^	0.509
		1.00 (Ref)	1.07 (0.72, 1.60)b1	0.89 (0.59, 1.36)b1	0.607
		1.00 (Ref)	1.19 (0.79, 1.78)c1	1.02 (0.66, 1.59)c1	0.879
Colon	991	1.00 (Ref)	0.98 (0.84, 1.13)^a^	0.83 (0.71, 0.97)^a^	0.018
		1.00 (Ref)	0.94 (0.81, 1.09)b1	0.82 (0.70, 0.96)b1	0.012
		1.00 (Ref)	0.98 (0.85, 1.15)c1	0.87 (0.74, 1.02)c1	0.083
**Rectum**	557	1.00 (Ref)	0.85 (0.70, 1.03)^a^	**0.71 (0.58, 0.87)**^a^, *	**<0.001**
		1.00 (Ref)	0.85 (0.70, 1.04)b1	**0.73 (0.60, 0.90)**b1, *	**<0.001**
		1.00 (Ref)	0.88 (0.72, 1.08)c1	**0.76 (0.61, 0.94)**c1, *	**0.010**
Liver	143	1.00 (Ref)	0.78 (0.53, 1.14)^a^	0.64 (0.42, 0.96)^a^	0.029
		1.00 (Ref)	0.78 (0.53, 1.14)b1	0.67 (0.44, 1.00)b1	0.047
		1.00 (Ref)	0.93 (0.63, 1.38)c1	0.85 (0.56, 1.31)c1	0.464
Pancreas	296	1.00 (Ref)	0.84 (0.63, 1.11)^a^	0.94 (0.72, 1.24)^a^	0.661
		1.00 (Ref)	0.80 (0.60, 1.06)b1	0.93 (0.71, 1.22)b1	0.581
		1.00 (Ref)	0.87 (0.65, 1.16)c1	1.05 (0.79, 1.40)c1	0.771
**Respiratory**	746	1.00 (Ref)	**0.64 (0.54, 0.75)**^a^, *	**0.59 (0.50, 0.70)**^a^, *	**<0.001**
		1.00 (Ref)	**0.60 (0.50, 0.71)**b1, *	**0.57 (0.48, 0.68)**b1, *	**<0.001**
		1.00 (Ref)	**0.74 (0.62, 0.88)**c1, *	**0.74 (0.61, 0.89)**c1, *	**<0.001**
**Lung**	705	1.00 (Ref)	**0.64 (0.53, 0.76)**^a^, *	**0.59 (0.49, 0.71)**^a^, *	**<0.001**
		1.00 (Ref)	**0.60 (0.50, 0.71)**b1, *	**0.56 (0.47, 0.68)**b1, *	**<0.001**
		1.00 (Ref)	**0.74 (0.62, 0.88)**c1, *	**0.73 (0.61, 0.88)**c1, *	**<0.001**
Mesothelial and soft tissue	193	1.00 (Ref)	1.15 (0.82, 1.60)^a^	0.81 (0.56, 1.16)^a^	0.275
		1.00 (Ref)	1.15 (0.82, 1.61)b1	0.84 (0.59, 1.22)b1	0.397
		1.00 (Ref)	1.16 (0.83, 1.62)c1	0.88 (0.61, 1.29)c1	0.559
Skin	5276	1.00 (Ref)	1.06 (0.99, 1.13)^a^	1.05 (0.98, 1.12)^a^	0.199
		1.00 (Ref)	1.02 (0.95, 1.09)b1	1.03 (0.97, 1.11)b1	0.327
		1.00 (Ref)	0.99 (0.93, 1.06)c1	1.00 (0.93, 1.07)c1	0.986
Melanoma	656	1.00 (Ref)	1.06 (0.88, 1.28)^a^	0.99 (0.82, 1.20)^a^	0.926
		1.00 (Ref)	1.03 (0.85, 1.24)b1	0.97 (0.80, 1.18)b1	0.772
		1.00 (Ref)	1.02 (0.85, 1.24)c1	0.98(0.80, 1.19)c1	0.824
Other skin	4620	1.00 (Ref)	1.06 (0.98, 1.13)^a^	1.05 (0.98, 1.13)^a^	0.159
		1.00 (Ref)	1.02 (0.95, 1.09)b1	1.04 (0.97, 1.12)b1	0.245
		1.00 (Ref)	0.99 (0.92, 1.06)c1	1.00 (0.93, 1.08)c1	0.911
Breast -female	2731	1.00 (Ref)	1.03 (0.93, 1.13)^a^	1.02 (0.93, 1.13)^a^	0.633
		1.00 (Ref)	1.01 (0.92, 1.11)b2	1.01 (0.92, 1.11)b2	0.877
		1.00 (Ref)	1.05 (0.95, 1.15)c2	1.04 (0.95, 1.15)c2	0.389
Female-genital organs	669	1.00 (Ref)	0.93 (0.77, 1.12)^a^	0.86 (0.71, 1.04)^a^	0.117
		1.00 (Ref)	0.89 (0.74, 1.08)b2	0.83 (0.69, 1.00)b2	0.055
		1.00 (Ref)	0.98 (0.81, 1.18)c2	0.93 (0.76, 1.13)c2	0.580
Uterus	356	1.00 (Ref)	0.92 (0.71, 1.18)^a^	0.87 (0.67, 1.13)^a^	0.293
		1.00 (Ref)	0.87 (0.67, 1.13)b2	0.84 (0.65, 1.08)b2	0.180
		1.00 (Ref)	1.00 (0.77, 1.30)c2	1.00 (0.76, 1.31)c2	0.890
Ovary	208	1.00 (Ref)	1.04 (0.75, 1.44)^a^	0.81 (0.58, 1.15)^a^	0.227
		1.00 (Ref)	1.00 (0.72, 1.40)b2	0.79 (0.56, 1.12)b2	0.171
		1.00 (Ref)	1.00 (0.71, 1.39)c2	0.78 (0.54, 1.12)c2	0.188
Male-genital organs	2966	1.00 (Ref)	1.16 (1.06, 1.26)^a^	1.16 (1.06, 1.27)^a^	<0.001
		1.00 (Ref)	1.06 (0.97, 1.16)b2	1.09 (1.00, 1.19)b2	0.050
		1.00 (Ref)	1.04 (0.95, 1.13)c3	1.04 (0.95, 1.14)c3	0.401
Prostate	2916	1.00 (Ref)	1.18 (1.08, 1.28)^a^	1.18 (1.08, 1.29)^a^	<0.001
		1.00 (Ref)	1.08 (0.99, 1.18)b2	1.10 (1.01, 1.21)b2	0.029
		1.00 (Ref)	1.05 (0.96, 1.15)c3	1.05 (0.96, 1.15)c3	0.284
Urinary organs	1028	1.00 (Ref)	1.00 (0.87, 1.16)^a^	0.83 (0.72, 0.97)^a^	0.023
		1.00 (Ref)	1.01 (0.88, 1.17)b1	0.89 (0.76, 1.04)b1	0.142
		1.00 (Ref)	1.12 (0.96, 1.30)c1	1.05 (0.89, 1.23)c1	0.520
Bladder	634	1.00 (Ref)	1.01 (0.84, 1.22)^a^	0.94 (0.78, 1.14)^a^	0.530
		1.00 (Ref)	1.03 (0.85, 1.24)b1	1.01 (0.83, 1.23)b1	0.911
		1.00 (Ref)	1.12 (0.92, 1.36)c1	1.16 (0.94, 1.41)c1	0.160
Kidney	394	1.00 (Ref)	0.99 (0.79, 1.24)^a^	0.68 (0.53, 0.88)^a^	0.004
		1.00 (Ref)	1.00 (0.79, 1.25)b1	0.71 (0.55, 0.92)b1	0.012
		1.00 (Ref)	1.12 (0.89, 1.42)c1	0.89 (0.68, 1.16)c1	0.464
Brain	253	1.00 (Ref)	0.91 (0.68, 1.22)^a^	0.80 (0.59, 1.08)^a^	0.148
		1.00 (Ref)	0.91 (0.67, 1.22)b1	0.82 (0.60, 1.12)b1	0.193
		1.00 (Ref)	0.94 (0.70, 1.27)c1	0.87 (0.64, 1.20)c1	0.402
**Thyroid**	104	1.00 (Ref)	**0.58 (0.37, 0.91)**^a^, *	**0.50 (0.31, 0.80)**^a^, *	**0.003**
		1.00 (Ref)	**0.55 (0.35, 0.86)**b1, *	**0.46 (0.28, 0.75)**b1, *	**<0.001**
		1.00 (Ref)	**0.53 (0.34, 0.85)**c1, *	**0.45 (0.27, 0.74)**c1, *	**<0.001**
Lymph	1236	1.00 (Ref)	0.95 (0.83, 1.08)^a^	0.93 (0.82, 1.07)^a^	0.330
		1.00 (Ref)	0.92 (0.80, 1.05)b1	0.93 (0.81, 1.07)b1	0.295
		1.00 (Ref)	0.93 (0.81, 1.07)c1	0.94 (0.81, 1.08)c1	0.382
Other	752	1.00 (Ref)	0.99 (0.83, 1.18)^a^	0.90 (0.75, 1.07)^a^	0.239
		1.00 (Ref)	0.94 (0.79, 1.12)b1	0.87 (0.73, 1.04)b1	0.119
		1.00 (Ref)	1.00 (0.84, 1.19)c1	0.95 (0.79, 1.15)c1	0.616

P for trend tests were conducted by including the median score of each IDI tertiles as a continuous variable in the models.

Bold values indicates p < 0.05.

a Unadjusted.

b1 Adjusted for age and sex.

b2 Adjusted for age.

c1 Adjusted for age, sex, race, education, Townsend deprivation index, energy intake, smoking, physical activity, body mass index, diabetes, hypertension, coronary artery disease, atrial fibrillation, heart failure, and stroke.

c2 Adjusted for age, race, education, Townsend deprivation index, energy intake, smoking, physical activity, body mass index, diabetes, hypertension, coronary artery disease, atrial fibrillation, heart failure, stroke, age at menarche, age at menopause, number of live births, oral contraceptive use, and use of hormone replacement therapy.

c3 Adjusted for age, race, education, Townsend deprivation index, energy intake, smoking, physical activity, body mass index, diabetes, hypertension, coronary artery disease, atrial fibrillation, heart failure, and stroke.

* p < 0.05.

Laplace regression analysis revealed that the multi-adjusted onset time of rectal cancer, thyroid cancer and lung cancer was delayed by 1.29 (10th PD, 95%CI: 0.32, 2.27) years, 2.62 (0.98, 4.27) years, 1.44 (0.58, 2.30) years for participants with low IDI scores, respectively, compared to those with high IDI scores (Table 3).

**Table 3:** 10th percentile differences (PDs) and 95% confidence intervals (CIs) of time at incident cancer (years) in relation to the inflammatory diet index (IDI).

Cancer	Number of cases	IDI-Categorical			P for trend
		High	Moderate	Low	
**Digestive**	2569	0.00 (Ref)	**0.58 (0.11, 1.05)**^a^, *	**1.34 (0.85, 1.83)**^a^, *	**<0.001**
		0.00 (Ref)	**0.68 (0.21, 1.15)**^b^, *	**1.28 (0.79, 1.76)**^b^, *	**<0.001**
		0.00 (Ref)	0.32 (−0.15, 0.80)^c^	**0.81 (0.31, 1.32)**^c^, *	**0.002**
**Rectum**	557	0.00 (Ref)	0.76 (−0.16, 1.68)^a^	**1.60 (0.63, 2.56)**^a^	**0.001**
		0.00 (Ref)	0.68 (−0.18, 1.54)^b^	**1.33 (0.44, 2.23)**^b^, *	**0.003**
		0.00 (Ref)	0.58 (−0.34, 1.50)^c^	**1.29 (0.32, 2.27)**^c^	**0.009**
**Respiratory**	746	0.00 (Ref)	**2.20 (1.36, 3.04)**^a^, *	**2.56 (1.70, 3.42)**^a^, *	**<0.001**
		0.00 (Ref)	**2.46 (1.64, 3.29)**^b^, *	**2.71 (1.87, 3.55)**^b^, *	**<0.001**
		0.00 (Ref)	**1.43 (0.60, 2.26)**^c^, *	**1.45 (0.58, 2.32)**^c^, *	**0.001**
**Lung**	705	0.00 (Ref)	**2.16 (1.30, 3.02)**^a^, *	**2.54 (1.66, 3.41)**^a^, *	**<0.001**
		0.00 (Ref)	**2.45 (1.60, 3.30)**^b^, *	**2.72 (1.86, 3.59)**^b^, *	**<0.001**
		0.00 (Ref)	**1.38 (0.56, 2.20)**^c^, *	**1.44 (0.58, 2.30)**^c^, *	**0.001**
**Thyroid**	104	0.00 (Ref)	**1.81 (0.27, 3.35)**^a^, *	**2.31 (0.72, 3.90)**^a^, *	**0.004**
		0.00 (Ref)	**1.99 (0.42, 3.55)**^b^, *	**2.53 (0.91, 4.15)**^b^, *	**0.002**
		0.00 (Ref)	**2.05 (0.49, 3.62)**^c^, *	**2.62 (0.98, 4.27)**^c^, *	**0.001**

P for trend tests were conducted by including the median score of each IDI tertiles as a continuous variable in the models.

Bold values indicates p < 0.05.

a Unadjusted.

b Adjusted for age and sex.

c Adjusted for age, sex, race, education, Townsend deprivation index, energy intake, smoking, physical activity, body mass index, diabetes, hypertension, coronary artery disease, atrial fibrillation, heart failure, and stroke.

* p < 0.05.

### 3.3 Stratified analyses for cancers by sex and age

**Fig. 2:**
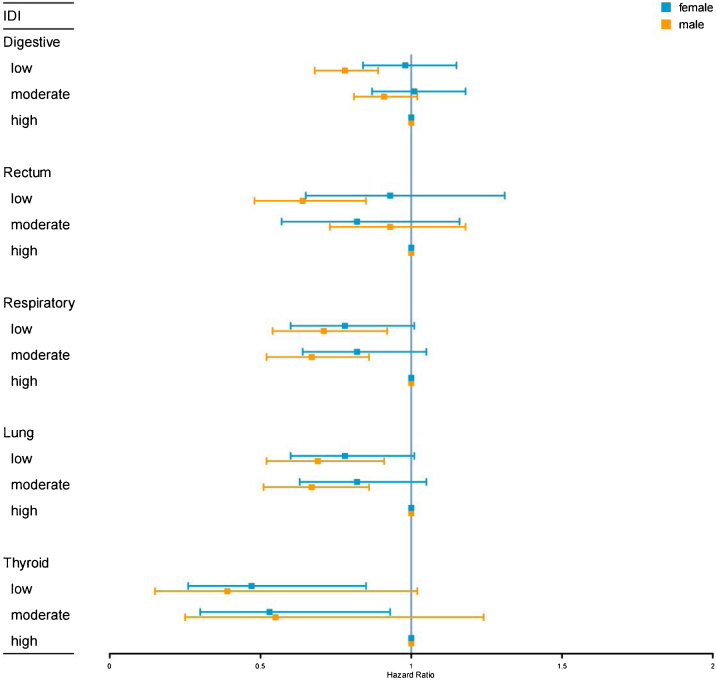
Hazard ratios (HRs) and 95% confidence intervals (CIs) of site-specific cancer in relation to the inflammatory diet index (IDI) stratified by gender after adjusting for age, race, education, Townsend deprivation index, energy intake, smoking, physical activity, body mass index, diabetes, hypertension, coronary artery disease, atrial fibrillation, heart failure, stroke, age at menarche, age at menopause, number of live births, oral contraceptive use, and use of hormone replacement therapy.

In multi-adjusted Cox regression, male participants with low IDI scores had risk reductions of rectal and lung cancer by 36% and 31%, respectively, compared to those with high IDI scores, while no association was found in females. There was only a significant multiplicative interaction between IDI score and sex for rectal cancer. However, female participants with low IDI scores had a lower risk of thyroid cancer compared to those with high IDI scores, while no association was found in males (Fig. 2 and Table S6).

**Fig. 3:**
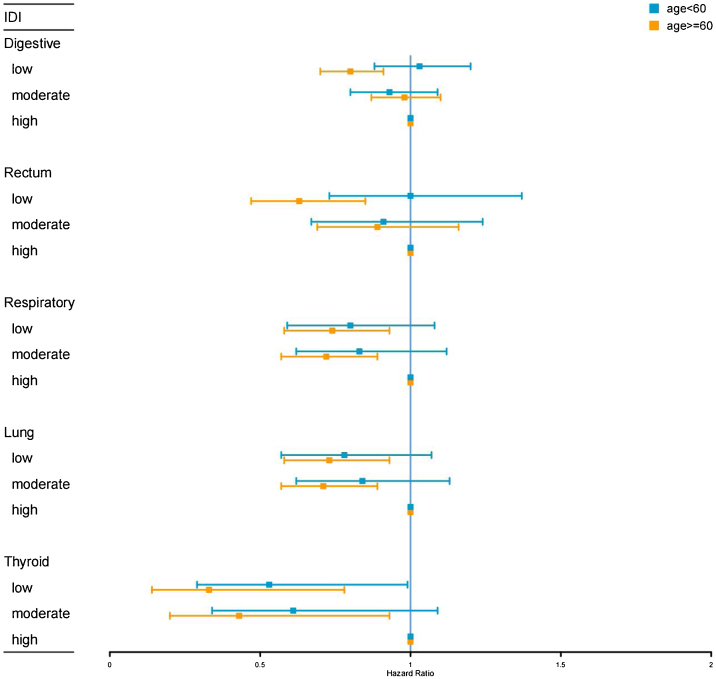
Hazard ratios (HRs) and 95% confidence intervals (CIs) of site-specific cancer in relation to the inflammatory diet index (IDI) stratified by age after adjusting for sex, race, education, Townsend deprivation index, energy intake, smoking, physical activity, body mass index, diabetes, hypertension, coronary artery disease, atrial fibrillation, heart failure, and stroke.

In multi-adjusted Cox regression, participants aged ≥60 with low IDI scores had a lower risk of rectal and lung cancer compared to those with high IDI scores, while no association was found in those aged <60. However, low IDI scores were associated with a decreased risk of thyroid cancer in participants aged <60 and those aged ≥60 (Fig. 3 and Table S7).

### 3.4 Supplementary analysis

We repeated the analyses after excluding 9878 (5.78%) participants with missing covariates (n = 161,021) (Table S8) and excluding incident cancer cases diagnosed within the first 2 years of follow-up (n = 167,847) (Table S9), and the results did not differ considerably from the main analyses.

## 4 Discussion

In this prospective study, we investigated the association of a low inflammatory diet with the risk of overall and site-specific cancers. We found that: 1) low IDI scores were associated with decreased risk of rectal cancer, thyroid cancer, and lung cancer, as well as prolonged onset time of these cancers compared with high IDI scores; 2) low IDI scores were associated with a lower risk of cancer in the rectum and lung in males, but not in females, and with a reduced risk of thyroid cancer in females, but not in males; and 3) low IDI scores were associated with a lower risk of rectal cancer and lung cancer in participants aged ≥60 years, but not in those aged <60.

An increasing number of studies suggest that chronic inflammation may play an important role in the development and progression of cancer [[29], [30], [31]]. There is also evidence to suggest that diet may contribute to chronic inflammation [[32], [33], [34]], indicating a potential for cancer risk reduction through dietary modification. Therefore, it is crucial to further investigate the relationship between dietary inflammatory potential and cancer risk.

At present, there are two common indices for evaluating dietary inflammation: the dietary inflammatory index (DII) and the empirical dietary inflammatory pattern (EDIP) score. The DII scoring is based on 45 pro-inflammatory and anti-inflammatory food parameters focusing on specific micronutrients and macronutrients rather than whole foods using a priori or investigator-driven approaches [35]. The EDIP is developed based on the relationship between18 food groups and circulating inflammatory markers (interleukin-6, C-reactive protein and tumor necrosis factor α receptor 2) in the US population using the hybrid method [36]. The IDI is calculated to assess the dietary inflammatory potential based on the relationship between 31 food groups and plasma high-sensitivity C-reactive protein in the UK Biobank study population.

The existing research on the association between a low-inflammatory diet and overall cancer have shown inconsistent results. In a meta-analysis examining the dose-response relationship of the dietary inflammatory index (DII) and cancer risk, including 44 high-quality studies (30 case-control studies, 14 cohort studies), overall cancer risk increased by 8.3% with each unit increase in DII score [6]. Another meta-analysis of the relationship between DII and cancer outcomes (13 case-control studies, six prospective cohort studies, one retrospective cohort study, three randomized controlled trials, and one unspecified study design) found that individuals in the highest DII category had a 25% increased risk of overall cancer compared to those in the lowest DII category [5]. However, two cohort studies (163,660 participants and 6542 participants, respectively) reported no association between a high-inflammatory diet (high DII scores) and overall cancer risk [11,12]. The present study did not found low IDI scores were associated with risk of overall cancer compared with high IDI scores. Discrepancies of research findings can be attributed to the different characteristics of study populations, sample sizes, possible confounders.

Few studies have analyzed the relationship between inflammatory properties of the diet and rectal cancer risk. In a case-control study in China (2502 cases and 2538 controls), the adjusted odds ratio (OR) for rectal cancer for participants in the highest versus lowest quartiles of the energy-adjusted DII (E-DII) was 1.53(8). Sex-stratified analysis showed that higher E-DII scores were significantly associated with the risk of rectal cancer only in men (OR_Q4 vsQ1_:1.74, 95% CI 1.35, 2.24). In addition, a cohort study with 26 years of follow-up (46,804 men and 74,246 women) similarly reported greater rectal cancer risk in relation to higher scores of empirical dietary inflammatory pattern (EDIP) scores in only men [14]. Another cohort study from Iowa Women’s Health Study showed no association between DII scores and rectal cancer in women [37]. Our findings in 68,135 males and 83,269 females suggest that a low-inflammatory diet may significantly reduce the risk of rectal cancer, especially in males. Males have a higher incidence of rectal cancer than females and are also more susceptible to dietary inflammation due to their unhealthier diets, both of which may be attributed to sex differences in biological and environmental risk factors [38,39]. For instance, males are more likely to consume more unhealthy food and consume more alcohol [40] and also tend to deposit more visceral fat [41], which is associated with an increased risk of rectal cancer in men [42]. Moreover, estrogen plays an essential protective role in the pathogenesis of rectal cancer in women, so menopause-related changes in estrogen levels and hormone replacement therapy may be potential confounders of the dietary inflammatory potential-rectal cancer association [43].

Currently, only a few studies have examined the relationship between inflammatory properties of the diet and lung cancer, and the results have been inconsistent. A case-control study in Iran (140 cases and 140 controls) found that odds of lung cancer of individuals with high DII scores was 2.01 times more than those with low DII scores, but this association was not significant in females [18]. Conversely, a cohort study from Australia with an average follow-up of 18 years (n = 35,303) found no significant difference in the risk of lung cancer between participants with low DII and high DII scores [16]. In current study, we found that a low-inflammatory diet was associated with a reduced risk of lung cancer, especially in males. Adherence to unhealthy lifestyles is more prevalent in men than in women [44]. Unhealthy behaviors as well as known or unknown environmental factors, such as occupational exposures and air pollution, could influence lung cancer alone or in conjunction with an inflammatory diet [45].

The association between inflammatory properties of the diet and thyroid cancer is rarely studied. A case-control study in New Caledonia (324 cases and 402 controls), revealed that participants with the highest DII scores had a 67% increased odds of thyroid cancer compared to those with low DII scores [46]. A cohort study from the European Prospective Investigation into Cancer and Nutrition (EPIC) found that for every 1-SD increase in DII score, participants had an 11% increased risk of differentiated thyroid cancer [47]. In agreement with these studies, we found that a low-inflammatory diet was related to a reduced risk of thyroid cancer, especially in females. Thyroid cancer is the only non-reproductive cancer with a significant female predominance [48], due to the promotion of thyroid cancer cells by estrogen specific to females [49].

Our results also suggest that a low-inflammatory diet may reduce the risk of rectal and trachea, bronchus, and lung cancers, especially in those ≥60 years of age. Some studies have shown that the immune system gradually erodes with age, and long-term chronic inflammation increases the incidence of cancer in the elderly [50]. While the aging process is irreversible, the state of chronic inflammation may be mitigated by dietary management [34], insinuating that the potential protective effect of a low-inflammatory diet may be more pronounced in this population.

The primary strength of our study is the large study sample and comprehensive assessment of the relationship between inflammatory properties of the diet and incidence of both site-specific and overall cancer. Nonetheless, some limitations should be acknowledged. First, UK Biobank participants were volunteers and may be healthier than the general population. This might have led to an underestimation of the association between dietary inflammatory potential and cancer risk. Second, 91.2% of participants were Caucasian, so caution is needed when extrapolating our findings to other ethnic groups. Third, due to the lack of the information on other inflammatory markers in the UK Biobank, we calculated the IDI score using only plasma hsCRP levels, which is a limited assessment of systemic inflammation levels. Fourth, our analyses do not capture the changes in dietary habits throughout follow-up, which could have led to inaccurate evaluations of dietary inflammatory potential. Firth, Due to the fewer number of cases of individual types of cancer, extrapolation of the results should still be done with caution. Finally, participants with undiagnosed cancer might have been misclassified as cancer-free, which could have led to misestimation of cancer risk.

## 5 Conclusion

A low-inflammatory diet is associated with a decreased risk of rectal cancer, lung cancer, and thyroid cancer, and may delay onset time of these cancers. Adhering to a low-inflammatory diet may be beneficial for preventing the development of rectal cancer and lung cancer, especially in males and older adults, and thyroid cancer especially in females.

## Author contributions

W.X. and X.Q. contributed to the conception and design of the study. J.L. and R.Y. conducted the statistical analyses, performed the literature search, and drafted the manuscript. H.D., J.W., M.M., M.M.D., X.Q., and W.X. reviewed and edited the manuscript. All authors critically revised the manuscript for important intellectual content. All authors made a significant contribution to finalize the manuscript and approved the final version for publication.

## Funding information

This work was supported by grants from the Swedish Research Council (No. 2021‐01647), the Swedish Council for Health Working Life and Welfare (2021‐01826), Karolinska Institutet Research Foundation (2022), the National Natural Science Foundation of China (No. 82204142), the scientific research project of 10.13039/501100010882Tianjin Municipal Education Commission (2021KJ118) and Tianjin Municipal Health Commission (2021057), China.

## Conflict of interest statement

The authors report no disclosures relevant to the manuscript.

## Data availability statement

Access to UK Biobank data can be requested through a standard data access procedure. Requests to access these datasets should be directed to http://www.ukbiobank.ac.uk/register-apply.

## Ethics statement

The UK Biobank study received ethical approval from the North West Multi-Centre Research Ethics Committee (Ref 11/NW/0382). All participants provided written informed consent, and all data used in this study were obtained from the UK Biobank (http://www.ukbiobank.ac.uk) through application 67048 (PI: Weili Xu).
