# Supplementary material for: The association of the dietary inflammatory potential with risk of overall and site-specific cancers: A community-based longitudinal study in the UK Biobank
Source: J Nutr Health Aging. 2024 Apr 5;28(6):100225. doi: 10.1016/j.jnha.2024.100225 (PMC12275773; doi:10.1016/j.jnha.2024.100225)
Supplement: Supplementary file 1 [file mmc1.docx]

**Supplementary materials**

**Table S1.** Cancer sites and the codes of the International Classification of Diseases, 9^th^ Revision (ICD9) and 10^th^ Revision (ICD10).

**Table S2.** Examples of food items constituting the 39 food groups (from the Oxford WebQ questionnaire of the UK Biobank).

**Table S3**. Factor loadings of reduced rank regression (RRR) dietary pattern.

**Table S4.** Foods included in the inflammatory diet index (IDI) and Spearman correlation coefficients between the individual food and plasma high-sensitivity C-reactive protein (hsCRP) concentrations.

**Table S5**. Hazard ratios (HRs) and 95% confidence intervals (CIs) of other site-specific cancer in relation to the inflammatory diet index (IDI).

**Table S6.** Hazard ratios (HRs) and 95% confidence intervals (CIs) of different forms of cancer in relation to inflammatory diet index (IDI) stratified by gender.

**Table S7.** Hazard ratios (HRs) and 95% confidence intervals (CIs) of different forms of cancer in relation to inflammatory diet index (IDI) stratified by age.

**Table** S**8.** Hazard ratios (HRs) and 95% confidence intervals (CIs) of different forms of cancer in relation to inflammatory diet index (IDI) after excluding participants with missing values for covariates (n=161,021).

**Table S9.** Hazard ratios (HRs) and 95% confidence intervals (CIs) of different forms of cancer in relation to inflammatory diet index (IDI) after excluding cancer cases diagnosed in the first 2 years of follow-up (n=167,847).

**Table S1.** Cancer sites and the codes of the International Classification of Diseases, 9^th^ Revision (ICD9) and 10^th^ Revision (ICD10).

| **Cancer sites** | **ICD10** | **ICD9** |
| --- | --- | --- |
| Lip and oral | C00-C08 | 140-145 |
| Pharynx | C09-C14 | 146-147 |
| Esophagus | C15 | 150 |
| Stomach | C16 | 151 |
| Small intestine | C17 | 152 |
| Colon | C18 | 153 |
| Rectum | C19-C20 | 1540,1541 |
| Anus | C21 | 1542,1543,1548 |
| Liver | C22 | 155 |
| Gall | C23-C24 | 156 |
| Pancreas | C25 | 157 |
| Other digestion | C26 | 158-159 |
| Nose | C30-C31 | 160 |
| Throat | C32 | 161 |
| Lung | C34 | 162 |
| Chest | C37-C39 | 163-169 |
| Bone | C40-C41 | 170 |
| Mesothelial and soft tissue | C45-C49 | 171 |
| Skin | C43-C44 | 172-173 |
| Breast | C50 | 174-175 |
| Cervix | C53 | 180 |
| Uterus | C54 | 182 |
| Ovary | C56 | 183 |
| Other female genital organs | C51,C52,C55,C57 | 179,184 |
| Testis | C62 | 186 |
| Penis | C60,C63 | 187 |
| Prostate | C61 | 185 |
| Bladder | C67 | 188 |
| Kidney | C64-C66,C68 | 189 |
| Eye | C69 | 190 |
| Brain | C70-C72 | 191-192 |
| Thyroid | C73 | 193 |
| Other gland | C74-C75 | 194 |
| Lymph | C81-C96 | 200-208 |
| Others | C76-C80,C97 | 195-199 |

**Table S2.** Examples of food items constituting the 39 food groups (from the Oxford WebQ questionnaire of the UK Biobank).

| **Food groups** | **Food items** |
| --- | --- |
| **Wine** | Red wine, rose wine, white wine, fortified wine |
| **Beer** | Beer/cider |
| **Other alcohol** | Spirits, other alcohol |
| **Bread** | Sliced bread, baguette, bap, bread roll, naan bread, garlic bread, crispbread, oatcakes, other bread |
| **Butter** | Butter/margarine |
| **Starch** | White pasta, wholemeal pasta, white rice, brown rice, sushi, snackpot, couscous, other grain |
| **Breakfast cereal** | Porridge, muesli, oat crunch, sweetened cereal, plain cereal, bran cereal, whole-wheat cereal, other cereal |
| **Bean** | Baked bean, pulses, broad bean, green bean, pea |
| **Potatoes** | Fried potatoes, boiled/baked potatoes, mashed potato |
| **Vegetables** | Mixed vegetable, vegetable pieces, coleslaw, side salad, avocado, beetroot, broccoli, butternut squash, cabbage/kale, carrot, cauliflower, celery, courgetti, cucumber, leek, lettuce, mushroom, onion, parsnip, sweet pepper, spinach, sprouts, sweetcorn, sweet potato, fresh tomato, tinned tomato, turnip/swede, watercress, other vegetables |
| **Garlic** | Garlic |
| **Fruit** | Stewed fruit, prune, dried fruit, mixed fruit, apple, banana, berry, cherry, grapefruit, grape, mango, melon, orange, satsuma, peach/nectarine, pear, pineapple, plum, other fruit |
| **Low-calorie drinks** | Low calorie drink |
| **High-calorie drinks** | Fizzy drink |
| **Juice** | Squash juice, orange juice, grapefruit juice, pure fruit/vegetable juice |
| **Smoothies** | Fruit smoothie, dairy smoothie |
| **Coffee** | Instant coffee, filtered coffee, cappuccino, latte, espresso, other coffee |
| **Tea** | Standard tea, rooibos tea, green tea, herbal tea, other tea |
| **Milk** | Milk, flavoured milk |
| **Other drink** | Other drink |
| **Processed meat** | Sausage, bacon, ham |
| **Red meat** | Beef, pork, lamb |
| **Poultry** | Crumbed or deep-fried poultry, poultry |
| **Organ meat** | Liver |
| **Other meat** | Other meat |
| **Fish** | Tinned tuna, oily fish, breaded fish, battered fish, white fish, prawns, Lobster/crab, shellfish, other fish |
| **Cheese** | Low fat hard cheese, hard cheese, soft cheese, blue cheese, low fat cheese spread, cheese spread, cottage cheese, feta, mozzarella, goat's cheese, other cheese |
| **Eggs** | Whole egg, omelette, eggs in sandwiches, scotch egg, other egg |
| **Pastry** | Double crust pastry, single crust pastry, crumble, pizza, pancake, scotch pancake, yorkshire pudding, indian snacks, croissant, danish pastry, scone |
| **Yogurt** | Yogurt |
| **Ice-cream** | Ice-cream |
| **Desserts** | Milk-based pudding, other milk-based pudding, soya dessert, fruitcake, cake, doughnut, sponge pudding, cheesecake, other dessert |
| **Chocolate** | Chocolate bar, white chocolate, milk chocolate, dark chocolate, chocolate-covered raisin, chocolate sweet, chocolate-covered biscuits, chocolate biscuits |
| **Chocolate drinks** | Low calorie hot chocolate, hot chocolate |
| **Sweets** | Diet sweets, sweets, sweet biscuits, cereal bar, other sweets |
| **Nuts** | Salted peanuts, unsalted peanuts, salted nuts, unsalted nuts, seeds |
| **Snack** | Crisp, savoury biscuits, cheesy biscuits, olives, other savoury snacks |
| **Soup** | Powdered/instant soup, canned soup, homemade soup |
| **Vegetarian protein alternatives** | Vegetarian sausages/burgers, tofu, quorn, other vegetarian alternatives |

**Table S3.** Factor loadings of reduced rank regression (RRR) dietary pattern.

| **RRR dietary pattern components^a^** | **Factor loadings** |
| --- | --- |
| **Positive associations** | |
| Low calorie drink | 0.0553 |
| Red meat | 0.0482 |
| Processed meat | 0.0350 |
| Poultry | 0.0268 |
| High calorie drink | 0.0262 |
| Egg | 0.0229 |
| Potato | 0.0200 |
| Butter | 0.0198 |
| Other alcohol | 0.0186 |
| Milk | 0.0171 |
| Ice-cream | 0.0137 |
| Organ meat | 0.0129 |
| Chocolate drink | 0.0107 |
| Sweet | 0.0084 |
| Other meat | 0.0052 |
| Smoothie | 0.0051 |
| Beer | 0.0047 |
| Snack | 0.0035 |
| **Inverse associations** | |
| Starch | -0.0423 |
| Nut | -0.0353 |
| wine | -0.0416 |
| Breakfast cereal | -0.0433 |
| Fruit | -0.0319 |
| Vegetable | -0.0189 |
| Bread | -0.0283 |
| Vegetarian protein alternative | -0.0161 |
| Fish | -0.0099 |
| Dessert | -0.0145 |
| Tea | -0.0109 |
| Pastry | -0.0064 |
| Cheese | -0.0130 |
| Juice | -0.0097 |
| Coffee | -0.0035 |
| Other drink | -0.0039 |
| Bean | -0.0030 |
| Chocolate | -0.0018 |
| Garlic | -0.0018 |
| Soup | -0.0019 |
| Yogurt | -0.0011 |

^a^ The RRR dietary pattern was the first factor obtained from RRR with all 39 food group.

**Table S4.** Food groups included in the inflammatory diet index (IDI) and Spearman correlation coefficients between the individual food group and high-sensitivity C-reactive protein (hsCRP) concentration.

| **Food Groups** | **Spearman correlation coefficients** | **Weights^a^** |
| --- | --- | --- |
| **Foods with anti-inflammatory potential** | |  |
| Wine | -0.06760579 | -0.00024999 |
| Bread | -0.00621024 | -0.00046442 |
| Starch | -0.06685762 | -0.00054338 |
| Breakfast | -0.07461232 | -0.00068301 |
| Vegetable | -0.04562040 | -0.00012807 |
| Fruit | -0.06292869 | -0.00019166 |
| Juice | -0.02393162 | -0.00004961 |
| Tea | -0.02319111 | -0.00002606 |
| Fish | -0.04415385 | -0.00021168 |
| Cheese | -0.03512007 | -0.00063409 |
| Pastry | -0.00390346 | -0.00010843 |
| Dessert | -0.01991112 | -0.00030916 |
| Nut | -0.06762819 | -0.00240000 |
| Vegetarian protein alternative | -0.04992702 | -0.00080500 |
| **Foods with pro-inflammatory potential** | |  |
| Beer | 0.00403657 | 0.00001233 |
| Other alcohol | 0.01599994 | 0.00158000 |
| Butter | 0.02198379 | 0.00165000 |
| Potato | 0.04132457 | 0.00021352 |
| Low calorie drink | 0.07160512 | 0.00028370 |
| High calorie drink | 0.03589409 | 0.00022944 |
| Smoothie | 0.00277628 | 0.00007810 |
| Milk | 0.01737686 | 0.00025221 |
| Processed meat | 0.04377300 | 0.00123000 |
| Red meat | 0.04949462 | 0.00093464 |
| poultry | 0.01287823 | 0.00051525 |
| Organ meat | 0.01072950 | 0.00168000 |
| Other meat | 0.00281912 | 0.00047258 |
| Egg | 0.02393672 | 0.00057291 |
| Ice cream | 0.01449329 | 0.00043748 |
| Sweet | 0.00088635 | 0.00039631 |
| Chocolate drink | 0.00689528 | 0.00026061 |

^a^ Weights are regression coefficients for each IDI component obtained from the last step of the stepwise linear regression analysis.

**Table S5**. Hazard ratios (HRs) and 95% confidence intervals (CIs) of other site-specific cancer in relation to the inflammatory diet index (IDI).

| Cancer | Number of cases | **IDI-Categorical** | | | **P for trend** |
| --- | --- | --- | --- | --- | --- |
|  |  | **High** | **Moderate** | **Low** |  |
| Pharynx | 77 | 1.00(Ref)  1.00(Ref)  1.00(Ref) | 0.76(0.44, 1.31) ^a^  0.84(0.49, 1.44) ^b1^  0.98(0.56, 1.70) ^c1^ | 0.79(0.46, 1.36) ^a^  0.90(0.52, 1.54) ^b1^  1.11(0.63, 1.94) ^c1^ | 0.380  0.679  0.738 |
| Small intestine | 58 | 1.00(Ref)  1.00(Ref)  1.00(Ref) | 1.14(0.63, 2.08) ^a^  1.09(0.60, 1.98) ^b1^  1.09(0.59, 2.01) ^c1^ | 0.74(0.38, 1.45) ^a^  0.73(0.37, 1.42) ^b1^  0.76(0.38, 1.53) ^c1^ | 0.410  0.370  0.466 |
| Anus | 48 | 1.00(Ref)  1.00(Ref)  1.00(Ref) | 1.14(0.56, 2.33) ^a^  1.06(0.51, 2.17) ^b1^  1.02(0.49, 2.11) ^c1^ | 1.27(0.63, 2.56) ^a^  1.18(0.59, 2.38) ^b1^  1.00(0.48, 2.08) ^c1^ | 0.500  0.638  1.000 |
| Gall | 58 | 1.00(Ref)  1.00(Ref)  1.00(Ref) | 0.55(0.29, 1.04) ^a^  **0.52(0.28, 0.98) ^b1^**  0.61(0.32, 1.16) ^c1^ | 0.59(0.32, 1.09) ^a^  0.57(0.31, 1.06) ^b1^  0.71(0.37, 1.36) ^c1^ | 0.073  0.059  0.252 |
| Other-digestive organs | 11 | 1.00(Ref)  1.00(Ref)  1.00(Ref) | 1.32(0.30, 5.91) ^a^  1.28(0.29, 5.77) ^b1^  1.39(0.30, 6.34) ^c1^ | 1.32(0.30, 5.89) ^a^  1.29(0.29, 5.81) ^b1^  1.53(0.32, 7.24) ^c1^ | 0.722  0.745  0.596 |
| Nose | 16 | 1.00(Ref)  1.00(Ref)  1.00(Ref) | 0.71(0.23, 2.24) ^a^  0.70(0.22, 2.21) ^b1^  0.79(0.24, 2.54) ^c1^ | 0.57(0.17, 1.93) ^a^  0.57(0.17, 1.95) ^b1^  0.65(0.18, 2.35) ^c1^ | 0.352  0.356  0.505 |
| Throat | 25 | 1.00(Ref)  1.00(Ref)  1.00(Ref) | 0.50(0.19, 1.32) ^a^  0.53(0.20, 1.41) ^b1^  0.71(0.26, 1.92) ^c1^ | 0.58(0.23, 1.47) ^a^  0.67(0.26, 1.69) ^b1^  1.05(0.40, 2.80) ^c1^ | 0.213  0.336  0.981 |
| Chest | 8 | 1.00(Ref)  1.00(Ref)  1.00(Ref) | 4.98(0.58, 42.59) ^a^  4.69(0.55, 40.35) ^b1^  4.19(1.00, 17.52) ^c1^ | 1.98(0.18, 21.85) ^a^  1.92(0.17, 21.32) ^b1^  1.90(0.38, 9.40) ^c1^ | 0.661  0.689  0.691 |
| Bone | 27 | 1.00(Ref)  1.00(Ref)  1.00(Ref) | 0.91(0.39, 2.14) ^a^  0.93(0.39, 2.20) ^b1^  0.94(0.39, 2.26) ^c1^ | 0.54(0.20, 1.46) ^a^  0.57(0.21, 1.56) ^b1^  0.60(0.21, 1.70) ^c1^ | 0.236  0.288  0.356 |
| Breast- male | 23 | 1.00(Ref)  1.00(Ref)  1.00(Ref) | 0.84(0.32, 2.21) ^a^  0.77(0.29, 2.02) ^b2^  0.85(0.32, 2.27) ^c3^ | 0.77(0.28, 2.11) ^a^  0.72(0.26, 1.97) ^b2^  0.80(0.28, 2.30) ^c3^ | 0.594  0.499  0.666 |
| Cervix | 34 | 1.00(Ref)  1.00(Ref)  1.00(Ref) | 1.12(0.47, 2.66) ^a^  1.12(0.47, 2.67) ^b2^  1.39(0.57, 3.34) ^c2^ | 1.16(0.49, 2.70) ^a^  1.15(0.49, 2.70) ^b2^  1.56(0.64, 3.79) ^c2^ | 0.745  0.749  0.319 |
| Other female  genital organs | 71 | 1.00(Ref)  1.00(Ref)  1.00(Ref) | 0.67(0.37, 1.21) ^a^  0.64(0.35, 1.15) ^b2^  0.66(0.37, 1.21) ^c2^ | 0.83(0.48, 1.44) ^a^  0.80(0.46, 1.38) ^b2^  0.85(0.48, 1.51) ^c2^ | 0.533  0.445  0.576 |
| Testis | 33 | 1.00(Ref)  1.00(Ref)  1.00(Ref) | **0.33(0.12, 0.90) ^a^**  **0.36(0.13, 0.98) ^b2^**  **0.36(0.13, 0.99) ^c3^** | 0.71(0.33, 1.54) ^a^  0.76(0.35, 1.65) ^b2^  0.67(0.30, 1.50) ^c3^ | 0.261  0.345  0.242 |
| Penis | 17 | 1.00(Ref)  1.00(Ref)  1.00(Ref) | 0.54(0.16, 1.74) ^a^  0.49(0.15, 1.60) ^b2^  0.52(0.16, 1.72) ^c3^ | 0.57(0.17, 1.84) ^a^  0.53(0.16, 1.73) ^b2^  0.50(0.15, 1.70) ^c3^ | 0.291  0.240  0.229 |
| Eye | 26 | 1.00(Ref)  1.00(Ref)  1.00(Ref) | 0.43(0.16, 1.11) ^a^  0.42(0.16, 1.09) ^b1^  0.45(0.17, 1.20) ^c1^ | 0.42(0.16, 1.11) ^a^  0.42(0.16, 1.09) ^b1^  0.42(0.15, 1.14) ^c1^ | 0.056  0.052  0.068 |
| Other gland | 21 | 1.00(Ref)  1.00(Ref)  1.00(Ref) | 0.77(0.29, 2.08) ^a^  0.73(0.27, 1.97) ^b1^  0.73(0.26, 1.99) ^c1^ | 0.55(0.18, 1.64) ^a^  0.52(0.17, 1.57) ^b1^  0.48(0.15, 1.49) ^c1^ | 0.281  0.241  0.199 |

**^a^** Unadjusted. *p<0.05

**^b1^** Adjusted for age and sex and **^b2^** Adjusted for age.

**^c1^** Adjusted for age, sex, race, education, Townsend deprivation index, energy intake, smoking, physical activity, body mass index, diabetes, hypertension, coronary artery disease, atrial fibrillation, heart failure, and stroke.

**^c2^** Adjusted for age, race, education, Townsend deprivation index, energy intake, smoking, physical activity, body mass index, diabetes, hypertension, coronary artery disease, atrial fibrillation, heart failure, stroke, age at menarche, age at menopause, number of live births, oral contraceptive use, and use of hormone replacement therapy.

**^c3^** Adjusted for age, race, education, Townsend deprivation index, energy intake, smoking, physical activity, body mass index, diabetes, hypertension, coronary artery disease, atrial fibrillation, heart failure, and stroke.

P for trend tests were conducted by including the median score of each IDI tertiles as a continuous variable in the models.

**Table S6.** Hazard ratios (HRs) and 95% confidence intervals (CIs) of different forms of cancer in relation to inflammatory diet index (IDI) stratified by gender.

| Inflammatory diet index (IDI) | Female | | | Male | | |
| --- | --- | --- | --- | --- | --- | --- |
|  | No. of  Subjects | No. of  Cases | HR (95% CI) | No. of  Subjects | No. of  Cases | HR (95% CI) |
| **Digestive** |  |  |  |  |  |  |
| Low | 30766 | 373 | 0.99(0.85, 1.15) ^a^  0.94(0.81, 1.10) ^b^  0.98(0.84, 1.15) ^c1^ | 20839 | 373 | **0.72(0.64, 0.82) ^a^**  **0.68(0.60, 0.78) ^b^**  **0.78(0.68, 0.89) ^c2^** |
| Moderate | 29265 | 370 | 1.03(0.89, 1.20) ^a^  0.97(0.83, 1.13) ^b^  1.01(0.87, 1.18) ^c1^ | 22191 | 492 | 0.90(0.80, 1.01) ^a^  **0.83(0.74, 0.94) ^b^**  0.91(0.81, 1.02) ^c2^ |
| High | 24658 | 301 | 1.00 (Reference) | 26865 | 660 | 1.00 (Reference) |
| **Rectum** |  |  |  |  |  |  |
| Low | 30472 | 79 | 1.02(0.73, 1.42) ^a^  0.98(0.70, 1.37) ^b^  0.92(0.65, 1.31) ^c1^ | 20542 | 76 | **0.63(0.48, 0.82) ^a^**  **0.59(0.45, 0.78) ^b^**  **0.64(0.48, 0.85) ^c2^** |
| Moderate | 28958 | 63 | 0.85(0.60, 1.21) ^a^  0.82(0.57, 1.16) ^b^  0.82(0.57, 1.16) ^c1^ | 21821 | 122 | 0.95(0.75, 1.20) ^a^  0.89(0.70, 1.12) ^b^  0.93(0.73, 1.18) ^c2^ |
| High | 24419 | 62 | 1.00 (Reference) | 26360 | 155 | 1.00 (Reference) |
| **Respiratory** |  |  |  |  |  |  |
| Low | 30510 | 117 | **0.68(0.53, 0.87) ^a^**  **0.64(0.50, 0.82) ^b^**  0.78(0.60, 1.01) ^c1^ | 20548 | 82 | **0.53(0.41, 0.69) ^a^**  **0.50(0.39, 0.65) ^b^**  **0.71(0.54, 0.92) ^c2^** |
| Moderate | 29014 | 119 | **0.73(0.57, 0.93) ^a^**  **0.68(0.53, 0.87) ^b^**  0.82(0.64, 1.06) ^c1^ | 21793 | 94 | **0.58(0.45, 0.74) ^a^**  **0.53(0.41, 0.67) ^b^**  **0.67(0.52, 0.86) ^c2^** |
| High | 24495 | 138 | 1.00 (Reference) | 26401 | 196 | 1.00 (Reference) |
| **Lung** |  |  |  |  |  |  |
| Low | 30507 | 114 | **0.68(0.53, 0.87) ^a^**  **0.64(0.50, 0.82) ^b^**  0.78(0.60, 1.01) ^c1^ | 20540 | 74 | **0.53(0.40, 0.69) ^a^**  **0.49(0.37, 0.64) ^b^**  **0.69(0.52, 0.91) ^c2^** |
| Moderate | 29011 | 116 | **0.72(0.56, 0.93) ^a^**  **0.68(0.53, 0.87) ^b^**  0.82(0.64, 1.06) ^c1^ | 21785 | 86 | **0.58(0.45, 0.74) ^a^**  **0.52(0.41, 0.68) ^b^**  **0.67(0.51, 0.86) ^c2^** |
| High | 24492 | 135 | 1.00 (Reference) | 26385 | 180 | 1.00 (Reference) |
| **Thyroid** |  |  |  |  |  |  |
| Low | 30412 | 19 | **0.47(0.27, 0.84) ^a^**  **0.48(0.27, 0.85) ^b^**  **0.48(0.27, 0.88) ^c1^** | 20472 | 6 | 0.43(0.17, 1.07) ^a^  0.41(0.16, 1.04) ^b^  0.39(0.15, 1.02) ^c2^ |
| Moderate | 28915 | 20 | **0.53(0.30, 0.92) ^a^**  **0.53(0.30, 0.93) ^b^**  **0.54(0.30, 0.95) ^c1^** | 21708 | 9 | 0.60(0.27, 1.34) ^a^  0.58(0.26, 1.28) ^b^  0.55(0.25, 1.24) ^c2^ |
| High | 24389 | 32 | 1.00 (Reference) | 26223 | 18 | 1.00 (Reference) |

^a^ Unadjusted and  ^b^ Adjusted for age.

^c1^ Adjusted for age, race, education, Townsend deprivation index, energy intake, smoking, physical activity, body mass index, diabetes, hypertension, coronary artery disease, atrial fibrillation, heart failure, stroke, age at menarche, age at menopause, number of live births, oral contraceptive use, use of hormone replacement therapy.

^c2^ Adjusted for age, race, education, Townsend deprivation index, energy intake, smoking, physical activity, body mass index, diabetes, hypertension, coronary artery disease, atrial fibrillation, heart failure, stroke.

**Table S7.** Hazard ratios (HRs) and 95% confidence intervals (CIs) of different forms of cancer in relation to inflammatory diet index (IDI) stratified by age.

| Inflammatory diet index (IDI) | Age<60 years | | | Age≥60 years | | |
| --- | --- | --- | --- | --- | --- | --- |
|  | No. of  Subjects | No. of  Cases | HR (95% CI) | No. of  Subjects | No. of  Cases | HR (95% CI) |
| **Digestive** |  |  |  |  |  |  |
| Low | 32602 | 314 | **0.84(0.72, 0.97) ^a^**  0.89(0.77, 1.03) ^b^  1.03(0.88, 1.20) ^c^ | 19003 | 432 | **0.69(0.61, 0.79) ^a^**  **0.74(0.65, 0.84) ^b^**  **0.80(0.70, 0.91) ^c^** |
| Moderate | 31655 | 295 | **0.81(0.70, 0.94) ^a^**  **0.85(0.73, 0.99) ^b^**  0.93(0.80, 1.09) ^c^ | 19801 | 567 | **0.88(0.78, 0.99) ^a^**  0.92(0.82, 1.03) ^b^  0.98(0.87, 1.10) ^c^ |
| High | 33739 | 387 | 1.00 (Reference) | 17784 | 574 | 1.00 (Reference) |
| **Rectum** |  |  |  |  |  |  |
| Low | 32365 | 77 | 0.84(0.62, 1.13) ^a^  0.90(0.66, 1.21) ^b^  1.00(0.73, 1.37) ^c^ | 18649 | 78 | **0.59(0.44, 0.78) ^a^**  **0.64(0.48, 0.85) ^b^**  **0.63(0.47, 0.85) ^c^** |
| Moderate | 31433 | 73 | 0.81(0.60, 1.11) ^a^  0.86(0.63, 1.17) ^b^  0.91(0.67, 1.24) ^c^ | 19346 | 112 | 0.82(0.63, 1.06) ^a^  0.87(0.67, 1.13) ^b^  0.89(0.69, 1.16) ^c^ |
| High | 33447 | 95 | 1.00 (Reference) | 17332 | 122 | 1.00 (Reference) |
| **Respiratory** |  |  |  |  |  |  |
| Low | 32362 | 74 | **0.65(0.49, 0.87) ^a^**  **0.64(0.48, 0.86) ^b^**  0.80(0.59, 1.08) ^c^ | 18696 | 125 | **0.53(0.43, 0.66) ^a^**  **0.55(0.44, 0.68) ^b^**  **0.74(0.58, 0.93) ^c^** |
| Moderate | 31437 | 77 | **0.70(0.52, 0.93) ^a^**  **0.69(0.52, 0.92) ^b^**  0.83(0.62, 1.12) ^c^ | 19370 | 136 | **0.56(0.45, 0.69) ^a^**  **0.57(0.46, 0.71) ^b^**  **0.72(0.57, 0.89) ^c^** |
| High | 33469 | 117 | 1.00 (Reference) | 17427 | 217 | 1.00 (Reference) |
| **Lung** |  |  |  |  |  |  |
| Low | 32358 | 70 | **0.65(0.48, 0.88) ^a^**  **0.63(0.47, 0.86) ^b^**  0.78(0.57, 1.07) ^c^ | 18689 | 118 | **0.53(0.42, 0.67) ^a^**  **0.54(0.43, 0.68) ^b^**  **0.73(0.58, 0.93) ^c^** |
| Moderate | 31434 | 74 | **0.71(0.53, 0.95) ^a^**  **0.69(0.52, 0.93) ^b^**  0.84(0.62, 1.13) ^c^ | 19362 | 128 | **0.56(0.45, 0.70) ^a^**  **0.57(0.45, 0.71) ^b^**  **0.71(0.57, 0.89) ^c^** |
| High | 33463 | 111 | 1.00 (Reference) | 17414 | 204 | 1.00 (Reference) |
| **Thyroid** |  |  |  |  |  |  |
| Low | 32305 | 17 | 0.57(0.31, 1.02) ^a^  **0.51(0.28, 0.93) ^b^**  **0.53(0.29, 0.99) ^c^** | 18579 | 8 | **0.39(0.17, 0.89) ^a^**  **0.38(0.17, 0.87) ^b^**  **0.33(0.14, 0.78) ^c^** |
| Moderate | 31379 | 19 | 0.65(0.37, 1.15) ^a^  0.60(0.34, 1.06) ^b^  0.61(0.34, 1.09) ^c^ | 19244 | 10 | **0.47(0.22, 1.01) ^a^**  **0.46(0.21, 1.00) ^b^**  **0.43(0.20, 0.93) ^c^** |
| High | 33383 | 31 | 1.00 (Reference) | 17229 | 19 | 1.00 (Reference) |

^a^ Unadjusted and ^b^ Adjusted for sex.

^c^ Adjusted for sex, race, education, Townsend deprivation index, energy intake, smoking, physical activity, body mass index, diabetes, hypertension, coronary artery disease, atrial fibrillation, heart failure, stroke.

**Table S8.** Hazard ratios (HRs) and 95% confidence intervals (CIs) of different forms of cancer in relation to inflammatory diet index (IDI) after excluding participants with missing values for covariates (n=161,021).

| Cancer | Number of cases |  | **IDI-Categorical** | | **P for trend** |
| --- | --- | --- | --- | --- | --- |
|  |  | **High** | **Moderate** | **Low** |  |
| **Digestive** | 2405 | 1.00(Ref)  1.00(Ref)  1.00(Ref) | 0.94(0.86, 1.04) ^a^  0.92(0.84, 1.01) ^b1^  0.99(0.90, 1.09) ^c1^ | **0.81(0.73, 0.90) ^a*^**  **0.82(0.74, 0.90) ^b1*^**  **0.90(0.81, 1.00) ^c1*^** | **<0.001**  **<0.001**  0.051 |
| **Rectum** | 522 | 1.00(Ref)  1.00(Ref)  1.00(Ref) | 0.90(0.73, 1.10) ^a^  0.90(0.73, 1.10) ^b1^  0.93(0.75, 1.14) ^c1^ | **0.76(0.61, 0.94) ^a*^**  **0.79(0.63, 0.97) ^b1*^**  0.81(0.65, 1.01) ^c1^ | **0.011**  **0.027**  0.060 |
| **Respiratory** | 681 | 1.00(Ref)  1.00(Ref)  1.00(Ref) | **0.64(0.54, 0.77) ^a*^**  **0.60(0.50, 0.72) ^b1*^**  **0.75(0.63, 0.90) ^c1*^** | **0.62(0.52, 0.75) ^a*^**  **0.60(0.50, 0.72) ^b1*^**  **0.78(0.65, 0.95) ^c1*^** | **<0.001**  **<0.001**  **0.006** |
| **Lung** | 641 | 1.00(Ref)  1.00(Ref)  1.00(Ref) | **0.65(0.54, 0.78) ^a*^**  **0.60(0.50, 0.73) ^b1*^**  **0.75(0.62, 0.91) ^c1*^** | **0.63(0.52, 0.76) ^a*^**  **0.60(0.50, 0.72) ^b1*^**  **0.78(0.64, 0.95) ^c1*^** | **<0.001**  **<0.001**  **0.008** |
| **Thyroid** | 100 | 1.00(Ref)  1.00(Ref)  1.00(Ref) | **0.55(0.34, 0.88) ^a*^**  **0.52(0.32, 0.83) ^b1*^**  **0.51(0.32, 0.83) ^c1*^** | **0.49(0.30, 0.79) ^a*^**  **0.45(0.28, 0.74) ^b1*^**  **0.45(0.27, 0.74) ^c1*^** | **0.002**  **<0.001**  **0.001** |

**^a^** Unadjusted.

**^b1^** Adjusted for age and sex.

**^c1^** Adjusted for age, sex, race, education, Townsend deprivation index, energy intake, smoking, physical activity, body mass index, diabetes, hypertension, coronary artery disease, atrial fibrillation, heart failure, and stroke.

P for trend tests were conducted by including the median score of each IDI tertiles as a continuous variable in the models. *p<0.05.

**Table S9.** Hazard ratios (HRs) and 95% confidence intervals (CIs) of different forms of cancer in relation to the inflammatory diet index (IDI) after excluding cancer cases diagnosed in the first 2 years of follw-up (n=167,847).

| Cancer | Number of cases | **IDI-Categorical** | | | **P for trend** |
| --- | --- | --- | --- | --- | --- |
|  |  | **High** | **Moderate** | **Low** |  |
| **Digestive** | 2145 | 1.00(Ref)  1.00(Ref)  1.00(Ref) | **0.88(0.80, 0.97) ^a*^**  **0.86(0.78, 0.95) ^b1*^**  0.93(0.84, 1.03) ^c1^ | **0.74(0.67, 0.83) ^a*^**  **0.75(0.68, 0.84) ^b1*^**  **0.83(0.74, 0.93) ^c1*^** | **<0.001**  **<0.001**  **<0.001** |
| **Rectum** | 466 | 1.00(Ref)  1.00(Ref)  1.00(Ref) | **0.80(0.64, 0.99) ^a*^**  **0.80(0.65, 0.99) ^b1*^**  0.83(0.67, 1.03) ^c1^ | **0.68(0.55, 0.86) ^a*^**  **0.71(0.56, 0.88) ^b1*^**  **0.72(0.57, 0.91) ^c1*^** | **<0.001**  **0.002**  **0.004** |
| **Respiratory** | 634 | 1.00(Ref)  1.00(Ref)  1.00(Ref) | **0.68(0.56, 0.82) ^a*^**  **0.64(0.53, 0.77) ^b1*^**  **0.79(0.65, 0.96) ^c1*^** | **0.61(0.50, 0.74) ^a*^**  **0.59(0.48, 0.71) ^b1*^**  **0.77(0.63, 0.94) ^c1*^** | **<0.001**  **<0.001**  **0.009** |
| **Lung** | 601 | 1.00(Ref)  1.00(Ref)  1.00(Ref) | **0.70(0.58, 0.84) ^a*^**  **0.65(0.54, 0.78) ^b1*^**  **0.81(0.66, 0.98) ^c1*^** | **0.62(0.51, 0.75) ^a*^**  **0.59(0.48, 0.71) ^b1*^**  **0.77(0.62, 0.94) ^c1*^** | **<0.001**  **<0.001**  **0.011** |
| **Thyroid** | 87 | 1.00(Ref)  1.00(Ref)  1.00(Ref) | **0.60(0.36, 0.99) ^a*^**  **0.57(0.34, 0.94) ^b1*^**  **0.57(0.34, 0.96) ^c1*^** | **0.57(0.34, 0.95) ^a*^**  **0.53(0.32, 0.89) ^b1*^**  **0.55(0.32, 0.94) ^c1*^** | **0.024**  **0.012**  **0.022** |

^a^ Unadjusted and ^b^ Adjusted for age and sex.

^c^ Adjusted for age, sex, race, education, Townsend deprivation index, energy intake, smoking, physical activity, body mass index, diabetes, hypertension, coronary artery disease, atrial fibrillation, heart failure, and stroke.

P for trend tests were conducted by including the median score of each IDI tertiles as a continuous variable in the models. **^*^**p<0.05.
